# Comparing efficacies of various papain-based enzyme agents and 2.4% sodium hypochlorite gel in chemomechanical caries removal: a randomized controlled trial

**DOI:** 10.1038/s41405-024-00258-9

**Published:** 2024-09-04

**Authors:** M. H. D. Baraa Alsayed, Mawia Karkoutly, Hassan Achour, Souad Abboud

**Affiliations:** 1 Department of Restorative Dentistry and Endodontics, Damascus University, Damascus, Syrian Arab Republic; 2 Department of Pediatric Dentistry, Damascus University, Damascus, Syrian Arab Republic

**Keywords:** Oral diseases, Restorative dentistry

## Abstract

**Objective:**

This study aimed to evaluate and compare the efficacies of Papacarie Duo gel, Brix 3000, Selecti-Solve gel, 2.4% sodium hypochlorite gel, and conventional rotary-mechanical method in caries removal and to evaluate the patient comfort.

**Methods:**

It was a single-blinded, randomized, parallel-group, active-controlled trial with five arms. It was conducted at the Department of Restorative Dentistry and Endodontics, Damascus University. Seventy-five specimens were randomly allocated into five groups: chemomechanical caries removal (CMCR) using Selecti-Solve gel (G1), BRIX3000 (G2), Papacarie DUO gel (G3), or 2.4% sodium hypochlorite gel (G4), and caries excavation using conventional rotary-mechanical method (G5) (control group). The trial considered healthy patients aged 18–40. Permanent molars with class I carious lesions extending to the middle third of dentin with no pulpal and/or periodontal pathology were included. The efficacy of caries removal was considered the primary outcome measure, and the secondary outcome measures were treatment time, volumetric measurement of the cavity, and pain assessment.

**Results:**

The majority (73.30%) of cases from BRIX3000 and conventional rotary-mechanical method groups showed complete caries removal (*p* = 0.982). The mean time of caries removal was the highest (17.45 ± 4.42) in the 2.4% sodium hypochlorite gel group (*p* < 0.05), and the lowest (6.33 ± 1.69) was in the conventional rotary-mechanical method group (*p* < 0.05). The mean cavity volume was the highest (18.97 ± 9.76) in the Papacarie DUO gel group, and the lowest (14.87 ± 4.76) was in the 2.4% sodium hypochlorite gel group (*p* = 0.506). The conventional rotary-mechanical group exhibited the highest mean score (5.40 ± 1.72) of pain (*p* < 0.05). However, the mean score (2.67 ± 1.11) of pain reported reduced in the BRIX3000 group.

**Conclusions:**

CMCR agents could be a potential substitute for conventional rotary instrumentation methods, taking into account the long working time.

## Introduction

Caries excavation using conventional rotary-mechanical methods is an invasive technique since it removes sound tooth structure and causes patient discomfort and adverse effects on dental pulp due to pressure, heat, vibration, and pain from high-speed drills. In addition, it requires local anesthesia. Therefore, although it is a time-efficient technique, it has several drawbacks, as outlined in previous studies [1]. The chemomechanical caries removal (CMCR) method was first introduced in the 1970s [2] and is a non-invasive and alternative technique that selectively eliminates infected dentine using various chemical agents to avoid patient discomfort and pulp irritation. CMCR dissolves infected tissue and preserves sound structure by applying synthetic or natural chemical agents followed by gentle excavation [3].

In 2011, Papacarie Duo gel was introduced as a CMCR agent based on papain enzyme [4]. Papacarie Duo gel contains papain enzyme, chloramine, and toluidine blue. Papain is a proteolytic enzyme extracted from the green papaya plant and has anti-inflammatory properties and increases partial degradation of type I collagen fibrils. In addition, the small amount of chloramine removes denatured issues [5]. Brix 3000 was recently introduced in 2016 as a modern modification of papain-based agents with papain concentration increased to 3000 U/mg in each 10% and encapsulated by encapsulated buffer emulsion (EBE) technology. EBE technology gives the optimal pH to enhance the degradation of collagen fibrils and provides better antimicrobial properties [6, 7]. In addition, Brix 3000 does not contain chloramine, which boosts its toxicological safety properties. However, the essential drawback of Brix 3000 is its higher cost [6, 7]. The selecti-Solve gel was first introduced in Egypt in 2021 as a cheaper papain-based agent, and it mainly consists of the papain enzyme, toluidine blue, and citrus pectin. However, Selecti-Solve gel has not been extensively study [8]. In addition, 2.25% sodium hypochlorite gel was used as a cheaper CMCR agent in primary teeth and yielded satisfactory outcomes in removing carious dentin, but its efficacy has not been studied in permanent teeth [6]. To the best of the authors’ knowledge, no study has compared the efficacies of the previous CMCR agents. Therefore, this study aimed to evaluate and compare the efficacies of Papacarie Duo gel, Brix 3000, Selecti-Solve gel, 2.4% sodium hypochlorite gel, and conventional rotary-mechanical method in caries removal and to evaluate the patient comfort.

## Materials and methods

### Study design

It was a single-blinded, randomized, parallel-group, active-controlled trial with five arms. It was conducted at the Department of Restorative Dentistry and Endodontics, Faculty of Dentistry, Damascus University, between February 2024 and April 2024. It was performed in full adherence to the CONSORT statement [9] and Declaration of Helsinki as revised in 2013 [10]. The study was approved by the Biomedical Research Ethics Committee at Damascus University (N2086), and the trial was registered on clinicalTrials.gov (NCT05733923) on 17/02/2024. Signed informed consent was provided from patients before enrollment.

### Study groups

The following parameters were considered to calculate the sample size: effect size of 0.41 (effect size f = 0.41), two-tailed 5% significance level (α err prob = 0.05), 95% confidence interval, 80% statistical power (1-β err prob = 0.80), and five experimental groups. The effect size was calculated according to a pilot study since the effect size was calculated by dividing the mean difference of the two samples by their standard deviation [11]. A sample size of seventy-five specimens was obtained. Eighty-three molars were assessed for eligibility, and seventy-five molars in sixty-seven patients were included according to the following inclusion and exclusion criteria: Inclusion criteria

1. Class I carious lesion in permanent first or second molar extending to the middle third of dentin.
2. Carious lesions are classified as ICDAS code 4.
3. Patient aged 18–40 years.
4. Cooperative patient accepting the trial.

### Exclusion criteria

1. Molars with pulpal and/or periodontal pathology.
2. Accidental pulp exposure during excavation.
3. Patient with medical complications.
4. Carious lesions with sclerotic dentin [12].

Seventy-five specimens were randomly allocated into five groups using online randomization software https://www.randomizer.org/. Grouping was as follows:

Group 1 (G1): CMCR using Selecti-Solve gel (SELECTI-SOLVE GEL, Denta Pharma, Cario, Egypt), *n* = 15.

Group 2 (G2): CMCR using BRIX3000 (BRIX3000®, BRIX Medical Science, Santa Fe, Argentina), *n* = 15.

Group 3 (G3): CMCR using Papacarie DUO gel (Papacarie® DUO, VARIOUS, São Paulo, Brazil), *n* = 15.

Group 4 (G4): CMCR using 2.4% sodium hypochlorite gel (Clorox® Toilet Bowl Cleaner - Clinging Bleach Gel, CLOROX, Oakland, United States), *n* = 15.

**Fig. 1:**
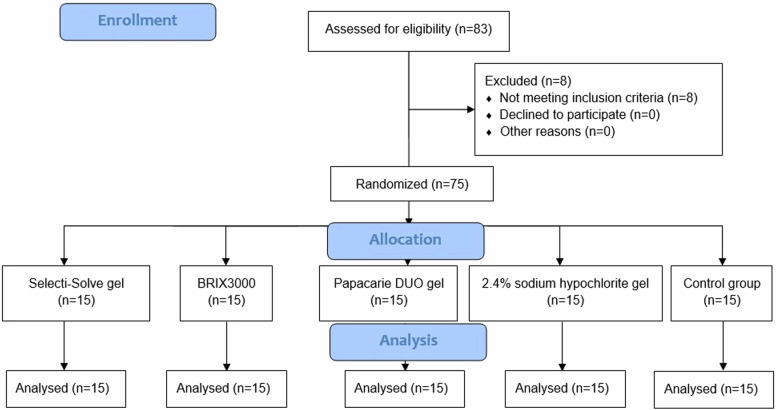
CONSORT flow diagram.

Group 5 (G5): Control group, caries excavation using conventional rotary-mechanical method, *n* = 15 (Fig. 1).

This was a single-blind trial where participants were masked to the group allocation.

### Intervention

A periapical X-ray radiograph was taken for each tooth by a mean of the intraoral periapical sensor (EzSensor HD, VATECH, Gyeonggi-do, Korea) before enrollment, and the tooth was isolated with a rubber dam. Unsupported enamel prisms were removed using an air turbine handpiece (NSK PANA-AIR, NSK Nakanishi Inc., Tochigi-ken, Japan) with copious irrigation. For CMCR agent groups, chemical agents were applied according to the manufacturer’s instructions, and then a sharp spoon excavator (17W, Medesy, Pordenone, Italy) was used to excavate carious dentin. Caries were excavated with the blunt back surface of the sharp spoon excavator utilizing rotational movements and light pressure. The procedure is repeated until the dentin has a hard texture and demonstrates no resistance. The dentin was checked using a sharp-tip dental probe (N.23/17A, Virco, Fareham, United Kingdom). For the conventional rotary-mechanical method group, the carious dentin was excavated using a round tungsten carbide bur (Excavabur E123A, Dentsply Maillefer, Ballaigues, Switzerland) in a contra-angle handpiece (NAC-EC, NSK Nakanishi Inc., Tochigi-ken, Japan). Cavities were restored with glass ionomer cement (GC Fuji IX GP®, GC America Inc., Illinois, United States) [6, 12].

### Primary outcome measures

#### Efficacy of caries removal

The efficacy of caries removal was evaluated using the Ericson et al. [13] scale by two blinded investigators. Cohen’s Kappa coefficient values of intra-examiner and inter-examiner reliability were >0.8. The examination of discoloration is visual and is followed by probing with a sharp probe to detect the texture of the dentin. The Ericson et al. scale is graded as follows:

0 = Complete removal of caries.

1 = Caries at the base of the cavity.

2 = Caries at the base of the cavity and/or the wall.

3 = Caries at the base of the cavity and/or two walls.

4 = Caries at the base of the cavity and/or more than two walls.

**Fig. 2:**
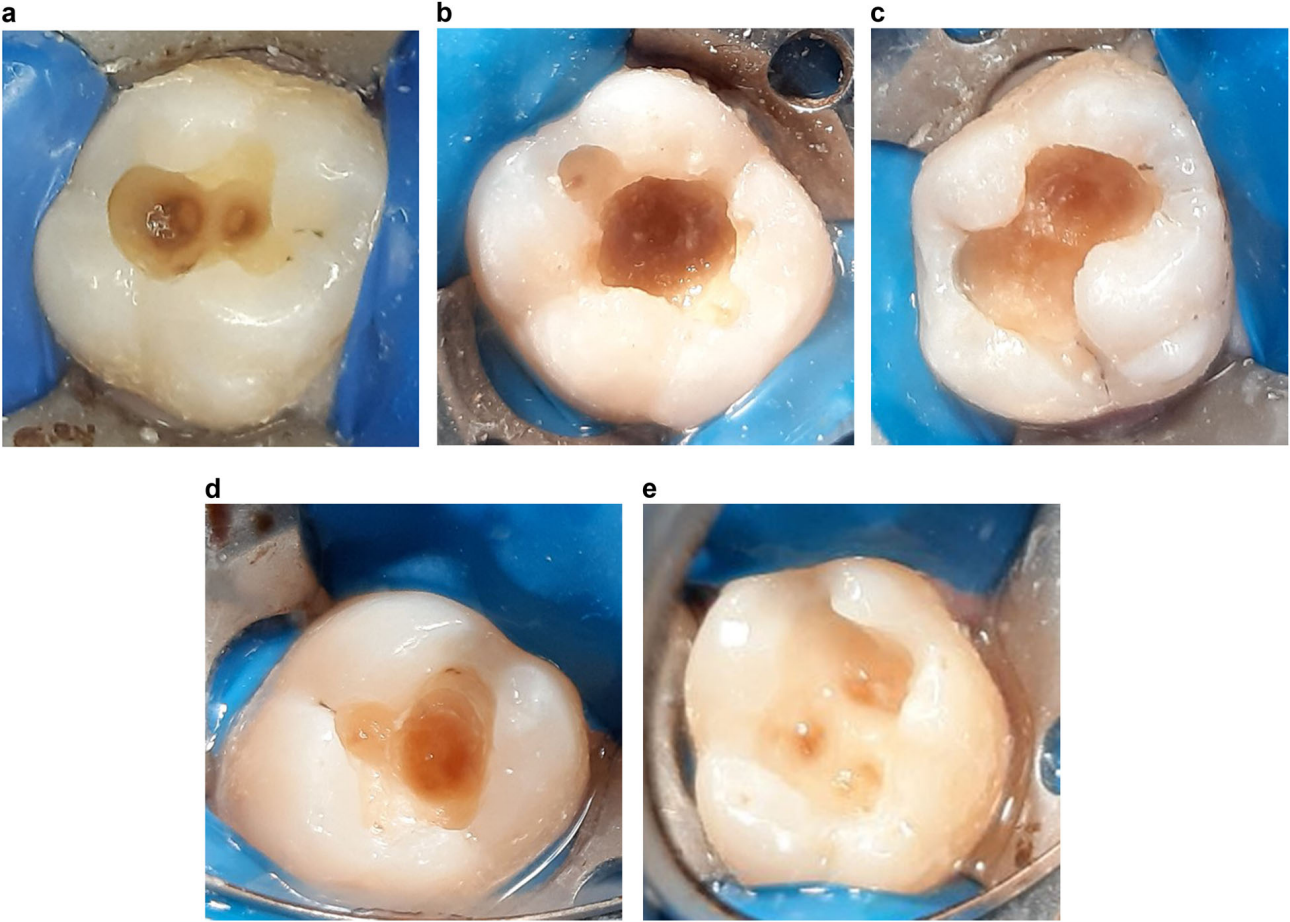
Cavities after caries excavation. **a** CMCR using Selecti-Solve gel. **b** CMCR using BRIX3000. **c** CMCR using Papacarie DUO gel. **d** CMCR using 2.4% sodium hypochlorite gel. **e** Caries excavation using conventional rotary instrumentation method.

5 = Caries at the base and the margins of the cavity and two walls (Fig. 2).

### Secondary outcome measures

#### Treatment time

The treatment time was determined utilizing a digital stopwatch. In the CMCR agents group, the calculation of treatment time started when first applying the chemical agent until it was confirmed that the cavity was free of caries. In the conventional rotary-mechanical method group, the calculation of treatment time started when first removing caries using round tungsten burs [6, 12].

#### Volumetric measurement of the cavity after excavation

**Fig. 3:**
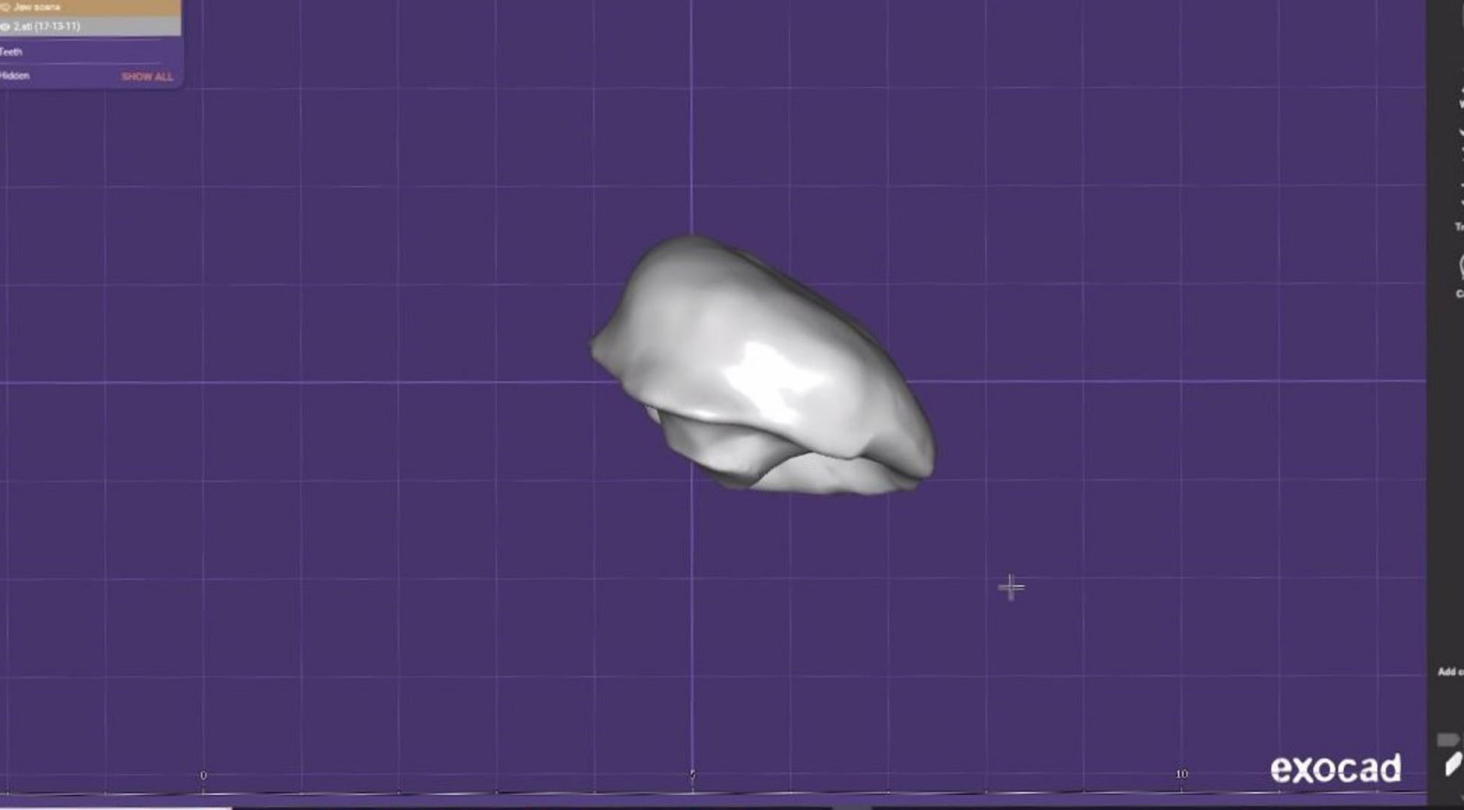
Volumetric measurement of the cavity after excavation utilizing exocad software.

A micro brush (MA-103, Threedental^TM^, New York, United States) was placed inside the prepared cavity, and the cavity was filled with gingival dam resin (FGM). It was light-cured utilizing a LED dental curing light (Power Led, Foshan Jerry Medical Apparatus Co., Ltd, Guangdong, China). The piece of resin was scanned using a scanner (Medit T710, Medit, Seoul, Korea). Subsequently, the exocad software (DentalCAD® 3.1 Rijeka, exocad, Hesse, Germany) was utilized to calculate the dimensions of the resin piece, which reflect the dimension of the prepared cavity (Fig. 3).

#### Pain assessment

The Numerical Rating Scale (NRS) was utilized to evaluate the pain intensity during caries excavation by a blinded investigator. The number (0) indicates the absence of pain during treatment, and the intensity of pain varies up to the number (5), which refers to moderate pain, and up to the number (10), which indicates very severe and unbearable pain [14].

### Statistical analysis

Data analysis was performed utilizing the IBM SPSS software version 26 (IBM Corp., Armonk, NY, USA). Data was presented as frequency, percentage, mean, standard deviation, standard error, minimum, and maximum. Kruskal–Wallis test and one-way ANOVA test were used to compare nonparametric and parametric data, respectively. The level of statistical significance was set at *p* < 0.05.

## Results

Approximately two-thirds (66%) of participants were female, and the mean age was 27.60 (SD 5.00; range 18–40) (Table 1). The results of efficacies of caries removal according to the Ericson et al. scale are listed in Table 2. The majority (73.30%) of cases from BRIX3000 and conventional rotary-mechanical method groups showed complete caries removal. However, only 6.70% of cases from the selecti-solve gel, BRIX3000, and 2.4% sodium hypochlorite gel groups showed caries at the base of the cavity and/or the wall with no statistically significant difference between groups (*p* = 0.982). The mean time of caries removal was the highest (17.45 ± 4.42) in the 2.4% sodium hypochlorite gel group, followed by the Selecti-Solve gel group (13.32 ± 3.19), BRIX3000 group (12.30 ± 2.93), Papacarie DUO gel group (11.92 ± 4.05), and the lowest (6.33 ± 1.69) was in conventional rotary-mechanical method group (Table 3), with a statistically significant difference between conventional rotary-mechanical group and other groups (*p* < 0.05), and 2.4% sodium hypochlorite gel group and other groups (*p* < 0.05) (Table 4). The mean cavity volume after excavation was the highest (18.97 ± 9.76) in the Papacarie DUO gel group, followed by the conventional rotary-mechanical group (16.80 ± 6.16), Selecti-Solve gel group (16.02 ± 5.22), BRIX3000 group (15.28 ± 7.20) and the lowest (14.87 ± 4.76) was in 2.4% sodium hypochlorite gel group with no statistically significant difference among groups (*p* = 0.506) (Table 5). The conventional rotary-mechanical group exhibited the highest mean score (5.40 ± 1.72) of NRS (Table 6) with a statistically significant difference to other groups (*p* < 0.05) (Table 6) followed by the Selecti-Solve gel group (3.27 ± 1.75), 2.4% sodium hypochlorite gel group (3.20 ± 1.21), and then Papacarie DUO gel group (3.13 ± 1.81). However, the mean score (2.67 ± 1.11) of pain reported reduced in the BRIX3000 group (Table 7).

**Table 1:** Demographic characteristics for each group.

Groups	*n*	Male		Female		Age	
		*n*	%	*n*	%	Mean	SD
Selecti-Solve gel	15	6	40.00	9	60.00	26.94	5.97
BRIX3000	15	6	40.00	9	60.00	30.06	12.10
Papacarie DUO gel	15	6	40.00	9	60.00	28.20	5.30
2.4% sodium hypochlorite gel	15	8	53.30	7	46.70	36.47	9.94
Conventional rotary-mechanical method	15	4	26.70	11	73.30	27.13	3.86
Total	75	30	40.00	45	60.00	27.60	5.00

**Table 2:** Results of Kruskal–Wallis test for comparing efficacies of caries removal according to Ericson et al. scale.

Scale	G1		G2		G3		G4		G5		Chi-square	*p*-value
	*n*	%	*n*	%	*n*	%	*n*	%	*n*	%		
0	10	66.70	11	73.30	10	66.70	10	66.70	11	73.30	0.407	0.982
1	4	26.70	3	20.00	5	33.33	4	26.70	4	26.70		
2	1	6.70	1	6.70	0	0.00	1	6.70	0	0.00		
3	0	0.00	0	0.00	0	0.00	0	0.00	0	0.00		
4	0	0.00	0	0.00	0	0.00	0	0.00	0	0.00		
5	0	0.00	0	0.00	0	0.00	0	0.00	0	0.00		
Mean rank	29.27		38.87		38.50		39.27		36.10			

*G1* Selecti-Solve gel, *G2* BRIX3000, *G3* Papacarie DUO gel, *G4* 2.4% sodium hypochlorite gel, *G5* Conventional rotary-mechanical method.

**Table 3:** Descriptive statistics and results of one-way ANOVA test for comparing treatment time.

Groups	Mean	SD	SE	Min	Max	F-value	*p*-value
G1	13.32	3.19	0.82	7.27	18.97	20.653	<0.001*
G2	12.30	2.93	0.76	6.40	17.87		
G3	11.92	4.05	1.04	5.92	19.08		
G4	17.45	4.42	1.14	9.65	23.35		
G5	6.33	1.69	0.44	3.00	8.70		

*G1* Selecti-Solve gel, *G2* BRIX3000, *G3* Papacarie DUO gel, *G4* 2.4% sodium hypochlorite gel, *G5* Conventional rotary-mechanical method.

*Significant difference at *p* < 0.05.

**Table 4:** Pairwise comparisons for comparing treatment time.

Pairwise comparisons	Mean difference	*p*-value
G1 vs. G2	1.01	1.000
G1 vs. G3	1.40	1.000
G1 vs. G4	−4.14	0.013*
G1 vs. G5	6.98	<0.001*
G2 vs. G3	0.39	1.000
G2 vs. G4	−5.15	0.001*
G2 vs. G5	5.97	<0.001*
G3 vs. G4	−5.54	<0.001*
G3 vs. G5	5.58	<0.001*
G4 vs. G5	11.12	<0.001*

*G1* Selecti-Solve gel, *G2* BRIX3000, *G3* Papacarie DUO gel, *G4* 2.4% sodium hypochlorite gel, *G5* Conventional rotary-mechanical method.

*Significant difference at *p* < 0.05.

**Table 5:** Descriptive statistics and results of one-way ANOVA test for comparing volumetric measures of the cavity after excavation.

Groups	Mean	SD	SE	Min	Max	F-value	*p*-value
G1	16.02	5.22	1.35	10.59	28.54	0.838	0.506
G2	15.28	7.20	1.86	5.64	30.79		
G3	18.97	9.76	2.52	11.09	42.06		
G4	14.87	4.76	1.23	7.42	23.07		
G5	16.80	6.16	1.59	9.61	33.61		

*G1* Selecti-Solve gel, *G2* BRIX3000, *G3* Papacarie DUO gel, *G4* 2.4% sodium hypochlorite gel, *G5* Conventional rotary-mechanical method.

**Table 6:** Pairwise comparisons for comparing NRS scores.

Pairwise comparisons	Mean difference	*p*-value
G1 vs. G2	0.60	0.393
G1 vs. G3	0.13	0.932
G1 vs. G4	0.07	0.797
G1 vs. G5	−2.13	0.003*
G2 vs. G3	−0.47	0.358
G2 vs. G4	−0.53	0.227
G2 vs. G5	−2.73	<0.001*
G3 vs. G4	−0.07	0.966
G3 vs. G5	−2.27	0.003*
G4 vs. G5	−2.20	0.001*

*G1* Selecti-Solve gel, *G2* BRIX3000, *G3* Papacarie DUO gel, *G4* 2.4% sodium hypochlorite gel, *G5* Conventional rotary-mechanical method.

*Significant difference at *p* < 0.05.

**Table 7:** Descriptive statistics and results of Kruskal–Wallis for comparing NRS scores.

Groups	Mean	SD	SE	Min	Max	Chi-square	*p*-value
G1	3.27	1.75	0.45	1	8	18.674	0.001*
G2	2.67	1.11	0.29	1	4		
G3	3.13	1.81	0.47	0	6		
G4	3.20	1.21	0.31	1	5		
G5	5.40	1.72	0.45	2	8		

*G1* Selecti-Solve gel, *G2* BRIX3000, *G3* Papacarie DUO gel, *G4* 2.4% sodium hypochlorite gel, *G5* Conventional rotary-mechanical method.

*Significant difference at *p* < 0.05.

## Discussion

Minimally invasive dentistry (MID) is a conservative approach that aims to maintain dental tissues and achieve patient comfort, and the CMCR method is a non-invasive technique that eliminates infected dentine via several chemical agents [1, 3]. According to Alkhouli et al. [6], sodium hypochlorite gel is an effective CMCR agent in removing carious lesions in primary teeth and was highly acceptable among pediatric patients. However, no study has ever evaluated the efficacy of sodium hypochlorite gel in caries removal in permanent teeth compared to other CMCR agents. Therefore, this study aimed to compare the efficacies of Papacarie Duo gel, Brix 3000, Selecti-Solve gel, 2.4% sodium hypochlorite gel, and conventional rotary-mechanical method in caries removal and to evaluate the patient comfort.

In this study, the conventional rotary-mechanical method was selected as a control group because Bastia et al. [12] suggested that the traditional rotary technique is highly acceptable among clinicians since it saves time and removes carious lesions effectively. The duration of application was one minute in the Papacarie DUO gel group and two minutes in the BRIX3000 gel and Selecti-Solve gel groups that is according to the manufacturer’s instructions. In addition, 2.4% sodium hypochlorite gel was applied for two minutes, which is similar to Alkhouli et al. [6] study. In the current study, the Ericson et al. [13] scale was utilized to evaluate the efficacy of caries removal due to its validity and acceptability. In addition, Assessment of the efficacy of caries removal was detected using a tactical method according to the Sadasiva et al. [15] study. Sadasiva et al. [15] suggested that the tactical method is similar to the efficacy of laser fluorescence and dye in detecting remaining caries. The NRS was used to subjectively evaluate pain intensity during caries excavation due to its simplicity and sensitivity to small changes in pain [14]. In the current study, a micro brush was placed inside the prepared cavity, and the cavity was filled with gingival dam resin, and then it was light-cured. The piece of resin was scanned utilizing a 3D scanner to conduct the volumetric measurement of the cavity after excavation. The previous technique saves time and achieves accuracy in terms of analyzing the cavity dimensions since it saves the unnecessary time required for making an impression and fabricating a cast [12].

The study found that the majority of cases from BRIX3000 gel showed complete caries removal. Similar results were reported by Gupta et al. [16], which suggested that BRIX3000 yielded satisfactory outcomes in caries removal in primary teeth. This could be attributed to the fact that BRIX3000 degrades collagen more efficiently since it has a high enzymatic activity compared to other CMCR agents [17]. In addition, papain concentration increased to 3000U/mg in each 10%, which adds to its efficacy [6]. The result of the current study suggested that the mean score of pain reported reduced in the BRIX3000 gel group, which is consistent with Alkhouli et al. [6] findings. Alkhouli et al. [6] suggested that BRIX3000 significantly reduced pain compared to the conventional rotary instrumentation method. Similarly, Batisa et al. [12] deduced that BRIX3000 was superior to rotary methods in controlling pain during excavation. The possible explanation for this finding is that BRIX3000 gel prevents the painful removal of caries and protects intact dentin since its efficacy is only restricted to demineralized dentin and denuded fibers [12, 16]. In addition, BRIX3000 yielded mild aesthetic effects during caries excavation. In addition, Bussadoriet al. [18] stated that papain gel can alleviate patient anxiety by removing caries effectively with the need for anesthesia. Papain-based agents have anti-inflammatory properties and operate only on the dead infected cells, which in turn cause a lower pain response [19]. Another possible explanation of this finding is that CMCR agents remove only infected dentin and preserve sound intact dentin, which is considered a painless procedure, according to the Mohanty et al. [20] study. The conventional rotary mechanical group exhibited the highest mean score of pain compared to the CMCR agent groups included in the current study. A possible explanation of this finding is that using a blunt spoon excavator for removing caries after applying CMCR agents reduces pressure and pain caused by conventional rotary instruments [2]. According to Kleinknech et al. [21], irritating procedures such as injection and drilling are not included in CMCR techniques. The finding of the current study is in agreement with Abdul et al. [22] and Ericson et al. [13] findings, which concluded that the CMCR method is more acceptable and comfortable. In addition, Goyal et al. [23] and Kochhar et al. [24] stated that pain significantly increases during caries excavation using rotary instruments compared to Papacarie DUO gel. However, conversely, Matsumoto et al. [25] stated that the pain experienced during caries excavation using Papacari DUO gel was comparable to that when using rotary instruments. In this study, there was no statistically significant difference in volumetric measures of the cavity after excavation between groups, indicating that cavity volume were standardized. In addition, all methods used to remove caries were conservative to a similar degree. However, the results in in contrast with the Batisa et al. [12] study, which deduced that BRIX3000 is highly conservative and preserves dentinal tissue when compared to the conventional rotary method. The current study showed that the mean time of caries removal was the lowest in the conventional rotary-mechanical method group, and the highest was in the 2.4% sodium hypochlorite gel group. This result is consistent with the Alkhouli et al. [6] study, which revealed that 2.25% sodium hypochlorite gel required 6.40 s compared to 1.60 s conventional-rotary instrumentation method in primary teeth. Although Carisolv is effective, according to many studies, as it contains 0.95% sodium hypochlorite solution, the sodium hypochlorite gel was not effective, according to the current study. Similarly, Dammaschke et al. [26] suggested that sodium hypochlorite gel is not as effective as cortisol in caries removal. The result of the current study is in agreement with Kitsahawong et al. [27] and Singh et al. [28] studies, which concluded that the conventional rotary-mechanical method required less time compared to the chemomechanical caries removal technique regardless of the chemical agents used. In addition, Batista et al. [12] study revealed that the time estimated for caries excavation using BRIX3000 is 2.5 min and 4.5 min for the rotary method. Similarly, according to Kochhar et al. [24], the minimum time for caries removal was taken by the conventional rotary method group. Kakaboura et al. [29] stated that the reason for the long working time when using CMCR agents is the demand for their multiple applications to get optimal efficacy. However, Matsumoto et al. [25] study demonstrated that the traditional method requires as much time as Papacarie DUO gel.

## Conclusions

Based on our findings, papain-based agents and 2.4% sodium hypochlorite gel are highly effective in caries removal and significantly reduce pain compared to the conventional rotary instrumentation method. However, the conventional rotary instrumentation method is more time-efficient compared to other CMCR agents. Both CMCR agents and conventional rotary instrumentation methods were conservative. Therefore, CMCR agents could be a potential substitute for conventional rotary instrumentation method with 2.4% sodium hypochlorite gel being the cheapest CMCR agent, taking into account the long working time. Further trials with a larger sample size recording patient-reported outcome measures are recommended to ascertain findings.

## Data Availability

The datasets generated during and/or analyzed during the current study are available from the corresponding author on reasonable request.
